# Effect of SiC Content on Microstructure and Mechanical Properties of CoCrFeNi High-Entropy Alloy Composites

**DOI:** 10.3390/ma19122501

**Published:** 2026-06-10

**Authors:** Ning Li, Xinlong Hu, Chengbo Wu, Mengyuan Jiang, Huiying Li, Jinlong Zhang, Fuyuan Dong

**Affiliations:** 1 School of Materials Science and Engineering, North Minzu University, Yinchuan 750021, China; wglining@163.com (N.L.); h482973218@163.com (X.H.); wcb030304@163.com (C.W.); jmengyuan2023@163.com (M.J.); 19805445697@163.com (H.L.); 19529282025@163.com (J.Z.); 2 National and Local Joint Engineering Research Center of Advanced Carbon-Based Ceramics Preparation Technology, Yinchuan 750021, China; 3 Key Laboratory of Powder Materials & Advanced Ceramics, Yinchuan 750021, China

**Keywords:** CoCrFeNi, SiC, spark plasma sintering, face-centered cubic, strengthening

## Abstract

In this work, to address the limitation of low strength and hardness of single-phase CoCrFeNi high-entropy alloy, SiC particles were introduced as a reinforcing phase to prepare CoCrFeNi matrix composites with SiC contents of 0 wt%, 1 wt%, 2.5 wt% and 5 wt% via spark plasma sintering (SPS). It was preliminarily predicted that SiC particles would be uniformly distributed along grain boundaries of the CoCrFeNi matrix. During sintering, partial SiC decomposes at high-temperature, high-activity interfaces, regulating carbide precipitation and phase structural evolution, while residual undecomposed SiC remains at grain boundaries to pin boundaries and refine grains, thereby synergistically enhancing mechanical properties and wear resistance. Microstructural characterization reveals that all samples maintain a face-centered cubic (FCC) solid-solution matrix, and samples with non-zero SiC addition contain Cr_7_C_3_ carbides, which are mostly distributed at grain boundaries. With the increase in SiC content, mechanical performance is remarkably improved compared with the unreinforced CoCrFeNi matrix: the hardness rises from 198.8 HV to 321.7 HV, the yield strength is greatly enhanced from 242.5 MPa to 673.4 MPa, and the tensile strength increases from 557.9 MPa to 755.7 MPa. The improved yield strength originates synergistically from grain refinement, solid-solution strengthening, grain-boundary strengthening and dislocation strengthening. By clarifying the influence of microstructural defects on critical shear stress (τ_0_) and normal fracture stress (σ_0_), the intrinsic mechanism governing tensile mechanical performance and ductile–brittle fracture transition was revealed. This optimized CoCrFeNi/SiC composite exhibits excellent strength–hardness comprehensive performance, showing promising application potential for high-load, wear-resistant and structural service components under severe tribological and pressure conditions.

## 1. Introduction

High-entropy alloys (HEAs), also referred to as multi-principal element alloys in some publications, have been widely studied in the metallic materials community in recent years, and the design concept of HEAs is to consist of multi-principal elements with iso- or near-isoatomic ratios. It was first proposed by Yeh and Cantor in 2004 [1,2]. While conventional alloys contain mainly one or two major elements, HEAs usually contain four or more major elements with atomic ratios of each element ranging from 5% to 35% [3]. The unique composition of HEAs provides excellent thermal stability [4], low-temperature mechanical properties [5] and corrosion resistance [6] by changing the elements and ratios of the constituent alloys. So far, most of the studies on HEAs have focused on the formation of single-phase structures [7,8,9,10,11,12,13,14,15], and among them CoCrFeNi, which has been used as one of the main studies, has relatively low strength at ambient temperatures. So despite the unique intrinsic characteristics of HEAs, such as high conformational entropy, slow atomic diffusion, severe lattice distortion and cocktail effect, there is a need to introduce other strengthening mechanisms to obtain competitive mechanical properties. In recent years, most of the strengthening methods applied to conventional alloys have been tried on high-alloyed steels, including biphasic strengthening [16,17], solid-solution strengthening [18,19], grain refinement strengthening [20,21] and precipitation strengthening [22,23].

The properties of HEAs can be greatly improved by adding some elements with larger atomic radii. For example, the addition of Nb/Ta to CoCrFeNi can achieve the effect of eutectic of FCC and Laves phases, which makes the alloy both ductile and strong [24]. In recent years, many studies have been conducted on the addition of ceramic particles to HEAs. For example, by adding C particles to the CoCrFeNi system, the yield strength can be significantly increased due to the presence of interstitial carbon atoms that hinder the dislocation motion and thus solid-solution strengthening. In addition, when the carbon content is higher than 0.3 wt%, a second phase is formed, which further strengthens the matrix [25]. Rogal et al. [26] added 5 wt% SiC particles to CoCrFeMnNi, and the compressive yield strength of the alloy was increased from 1180 MPa to 1480 MPa. Zhang et al. [27] added TiC to CoCrFeNi, and the mechanical properties of the alloy were also significantly improved. The effect of SiC on CoCrFeNiCu [28], Al_2_CoCrFeNi [29], and AlCoCrFeNi [30] has been previously investigated. However, few researchers have investigated the effect of SiC on CoCrFeNi substrates. In a word, conventional HEAs are composed of multiple principal elements with nearly equiatomic ratios, exhibiting unique lattice distortion, sluggish diffusion, high mixing entropy and cocktail effects. These superior characteristics provide HEAs with outstanding comprehensive performance, including high strength, excellent hardness, good corrosion resistance and favorable wear resistance, which make them promising candidates for advanced structural and functional materials.

In this study, we used a spark plasma sintering furnace for the sintering of metal matrix composites and the addition of silicon carbide (SiC) particles to the CoCrFeNi matrix to strengthen the alloy. Spark plasma sintering (SPS) is an efficient, rapid consolidation technology that integrates pulsed current heating, pressurized sintering and field-activated effect. Compared with conventional vacuum sintering and hot pressing, SPS features a fast heating rate, short holding time and low sintering temperature, which can effectively restrain abnormal grain growth, maintain fine microstructure, and facilitate interfacial reaction and densification of alloy matrix composites. The microstructure was analyzed and room-temperature tensile properties were measured. The fabricated samples showed excellent properties. The significant increase in strength can be attributed to the formation of various strengthening mechanisms, including diffusion strengthening, dislocation strengthening and grain-boundary strengthening. The effect of defects on the critical shear stress (τ_0_) and normal fracture stress (σ_0_) and the changes they cause to the fracture mode were analyzed.

The as-prepared CoCrFeNi/SiC composite exhibits excellent comprehensive mechanical properties, including high hardness, improved strength and favorable wear resistance, which provide it with promising industrial application potential. In the field of mechanical manufacturing, this material can be applied to produce wear-resistant molds, precision gears and cutting components serving under medium-load and friction conditions. In metallurgical and chemical industries, it is suitable for protective lining parts and conveying structural components working in high-temperature and corrosive environments. Furthermore, the composite can be fabricated via mature powder metallurgy and SPS sintering routes with controllable raw material cost and stable preparation reproducibility. The optimized composition of 0–5 wt% SiC enables adjustable strength–ductility matching, which makes it feasible for batch production and practical engineering promotion. Consequently, the CoCrFeNi/SiC composite possesses high application value and broad prospect in wearable structural parts, high-temperature resistant components and industrial equipment protection parts.

## 2. Experimental Materials and Methods

CoCrFeNi HEA powder prepared by aerosolization was used in this experiment; the particle size of the powder ranged from 15 μm to 53 μm, and the size of the SiC powder used was 500 nm. The CoCrFeNi HEA powder was mixed with 1 wt%, 2.5 wt%, and 5 wt% SiC powder for 8 h of ball milling and then sintered using a spark plasma sintering furnace (SPS-20T-10, Shanghai Chenhua Science and Technology Co., Ltd., Shanghai, China); for sintering, the sintering temperature and pressure were 1000 °C and 40 MPa, and the holding time was 5 min. The sintered samples were polished (the sintered samples were ground step by step using different grit silicon carbide paper and then mechanically polished with diamond polishing paste to obtain a smooth mirror surface) and processed for density determination by Archimedes drainage method [31].

**Figure 1:**
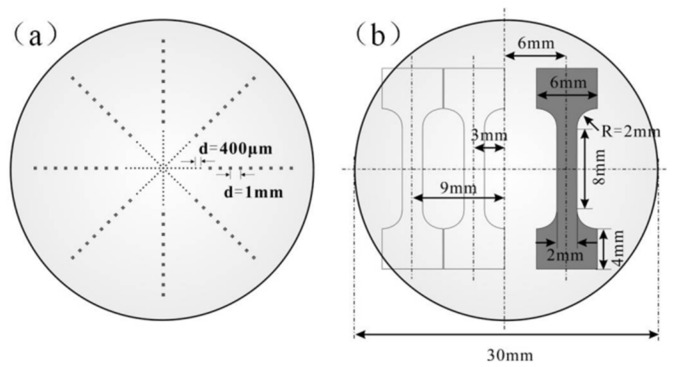
(**a**) The sintered sample and (**b**) the stretched sample. Reproduced with permission from [34].

**Figure 2:**
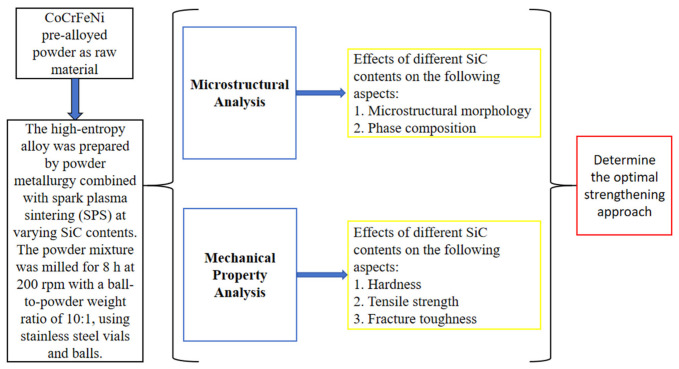
Overall flowchart.

The original samples were cut into dog-bone tensile samples and metallographic samples using a BM400-type center-wire cutting machine (Suzhou Baoma Numerical Control Equipment Co., Ltd., Suzhou, China), and the size of the tensile samples is shown in Figure 1. The gauge length of the dog-bone tensile specimens was ground with 2000-grit SiC abrasive paper, achieving a surface roughness of Ra ≈ 0.2–0.4 μm. Tensile tests were performed using a CMT5305 universal material testing machine (Universal testing machine, CMT5305, MTS Systems (China) Co., Ltd., Shanghai, China) to test the mechanical properties of the alloy, and the experiments were carried out at room temperature with a tensile rate of 1 × 10^−3^ mm/s. Three samples of each specimen were taken and stretched under the same process parameters. The Vickers microhardness test was carried out according to ISO 6507-2:2018 and ASTM E384-17 standards [32,33]; the hardness of the sintered samples at different SiC contents was measured using a micro Vickers hardness tester (HVS-1000, Laizhou Huaxing Testing Instruments Co., Ltd., Laizhou, China), and the indenter was made of diamond positive tetragonal prism with a load of 9.8 N and a duration of 10 s. In order to measure the microhardness of the alloys, 10 different positions in the samples were selected for measurement to ensure the accuracy of the results. Then, the maximum and minimum values of the data were removed, and the average value of the remaining data was taken as the microhardness of the sample. The phase composition was analyzed by X-ray diffractometry (XRD; XRD-6000 (3KW), Shimadzu (China) Co., Ltd., Shanghai, China) Cu target Kα radiation (tube voltage 40 kV, tube current 30 mA) with a scanning range of 20°~80° and a scanning speed of 4°/mm. Metallographic specimens sintered at 1000 °C were etched after grinding and polishing. Scanning electron microscopy and energy dispersive X-ray spectroscopy (SEM and EDS; Hitachi TM4000Plus II + Oxford X-Max, Hitachi (China) Ltd., Beijing, China) were used to observe the microstructure. Based on the SEM micrographs of CoCrFeNi alloys with different SiC contents, the intercept method, area method and point intercept method were adopted for grain size statistics. Several straight lines were randomly drawn on multiple micrographs of different fields of view (all grains in each SEM micrograph were labeled and statistically measured as comprehensively as possible), the number of grains intersected by the lines was counted, and the average grain size was calculated accordingly. The overall flowchart is shown in Figure 2.

## 3. Experimental Results and Analysis

### 3.1. Microstructure Analysis

**Figure 3:**
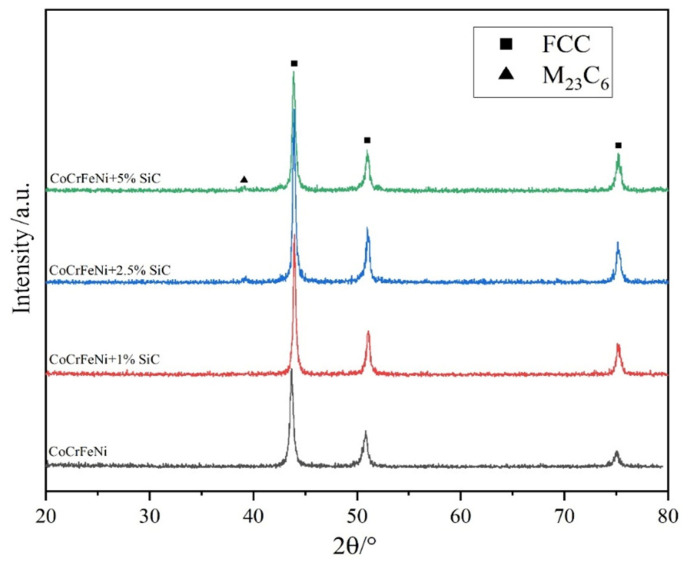
X-ray diffraction pattern.

Figure 3 shows the XRD spectra of the HEAs with the addition of 0, 1, 2.5, and 5 wt% SiC to CoCrFeNi (hereinafter referred to as samples S1–S4, respectively), from which it can be seen that the HEAs have only the FCC phase when the addition of SiC is 0; there are no other obvious diffraction peaks when the addition of SiC is 1%, which is probably due to the low content of SiC; and the diffraction peaks of S2 and S4 are shifted to the right compared with S1, which is caused by lattice distortion due to the addition of SiC, and the same phenomenon is also found in S3 and S4. With the gradual increase in SiC addition, the new phase, M_23_C_6_, appears in both S3 and S4. When transition elements are present, SiC is unstable at high temperatures and decomposes into Si and C elements that combine with metal elements in the alloy. Elemental combinations are determined by the mixing enthalpy, and when the mixing enthalpy is more negative, the elements are more likely to combine and agglomerate. The largest negative mixing enthalpy element pair in the sample is C-Cr, which is −61 KJ·mol^−1^, so the most stable carbide among them, Cr_7_C_3_, is formed after the reaction 7Cr + 3C → Cr_7_C_3_: ΔG = −136.6 KJ·mol^−1^ [35]. It forms a dense oxide-carbide film that serves as a solid lubricating layer to reduce the friction coefficient and improve wear stability. Accordingly, the precipitation strengthening mechanism induced by in situ carbides in tribological systems has been widely reported in SiC-reinforced CoCrFeNi composites. For instance, Zhang et al. [36] revealed that the decomposition of SiC during SPS leads to the formation of Cr_7_C_3_ and M_23_C_6_ carbides, which significantly improve the mechanical and tribological properties of the matrix.

**Figure 4:**
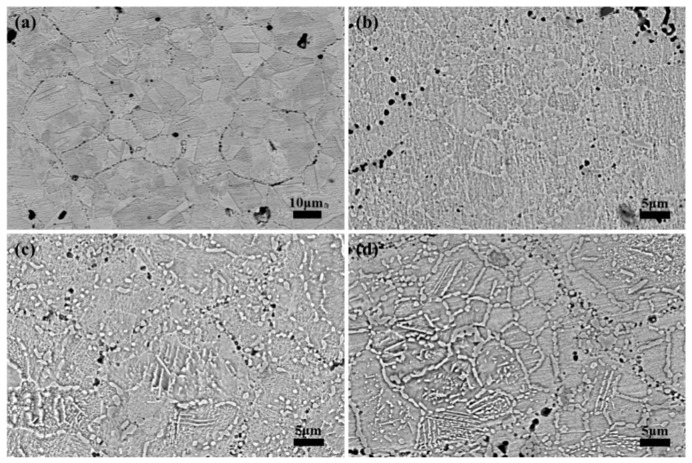
SEM of CoCrFeNi with different SiC contents: (**a**) 0%SiC. (**b**) 1%SiC. (**c**) 2.5%SiC. (**d**) 5%SiC.

**Figure 5:**
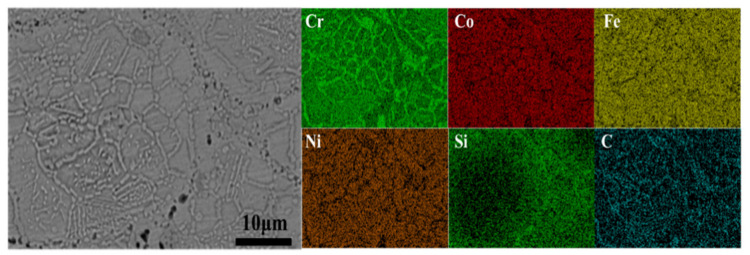
EDS diagram of CoCrFeNi with 5%SiC content.

Figure 4 shows the SEM images of samples S1–S4, in which due to the different SiC contents, the single-phase structure of CoCrFeNi HEA is transformed: when the SiC content is 0, the sample is a single-phase FCC structure, and when the SiC content is 1%, Cr_7_C_3_ precipitates out of the grain boundaries. As shown in Figure 4b, with the increase in SiC content, Cr_7_C_3_ precipitates at the grain boundaries, and precipitation obviously increases and gradually transforms from complete extended grain-boundary precipitation to incomplete continuous precipitation. In particular, when the SiC content is 5%, it is clearly observed that part of Cr_7_C_3_ parallel precipitation is no longer completely continuous and the grain boundaries are not completely continuous, but rather decomposition and aggregation can be observed, which is mainly due to the second solid phase partially wetting [37]. Figure 5 shows the EDS mapping analysis of S4, from which the Cr_7_C_3_ distribution can be clearly seen, due to the decomposition of SiC and the synthesis of Cr_7_C_3_, resulting in the aggregation of Si elements mainly in the FCC matrix.

**Figure 6:**
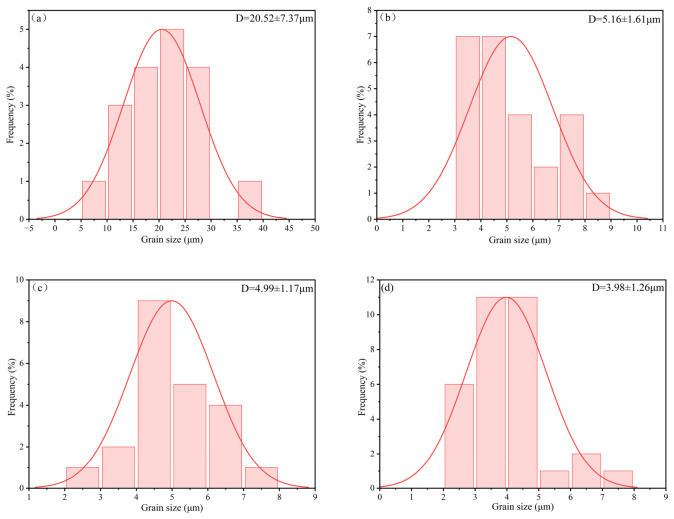
Grain size distribution histograms: (**a**) 0%SiC. (**b**) 1%SiC. (**c**) 2.5%SiC. (**d**) 5%SiC.

The grain size distribution histograms of each sample (0%, 1%, 2.5%, and 5%) are shown in Figure 6. It can be seen that the grain size decreases gradually with the increase in SiC content. A distinct difference in grain size is observed between the SiC-free sample and the 1% SiC-doped sample, which well explains the variation trend in the hardness curve.

### 3.2. Mechanical Properties Analysis

**Figure 7:**
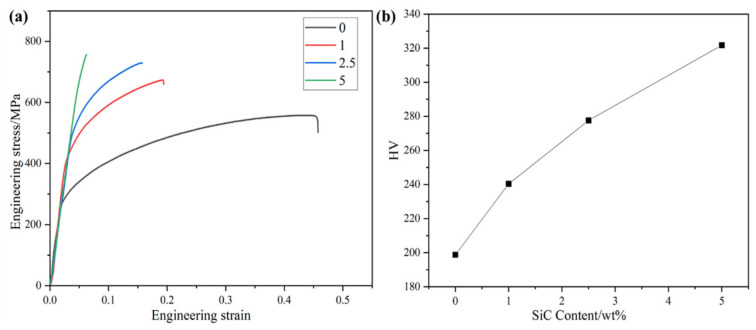
(**a**) Engineering stress–strain curve and (**b**) hardness variation diagram of samples with different SiC contents.

**Figure 8:**
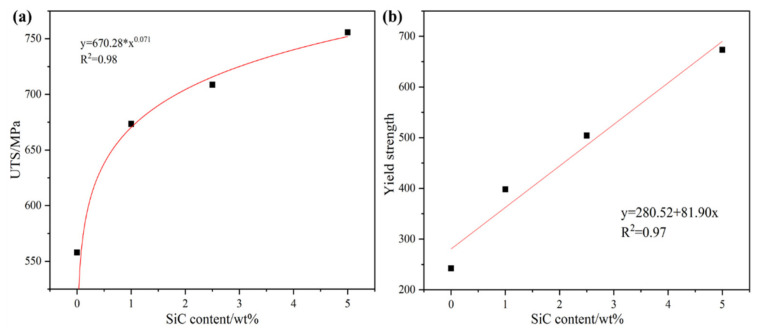
Trend chart of (**a**) ultimate tensile strength and (**b**) yield strength with different SiC contents.

**Figure 9:**
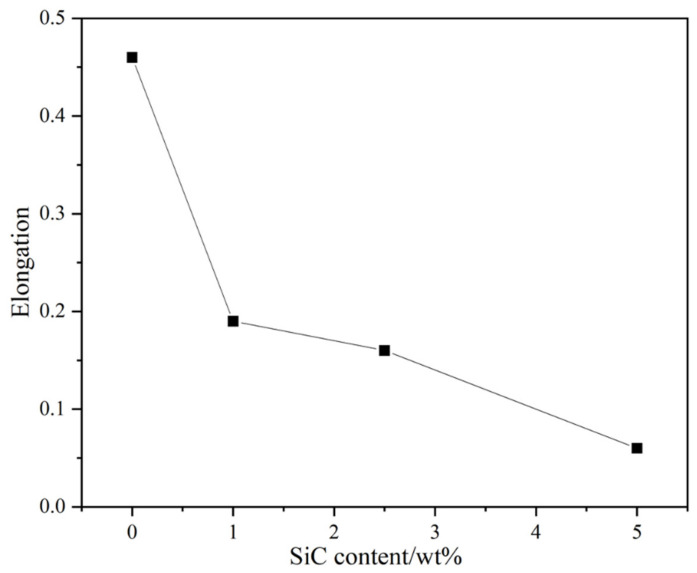
Elongation of samples with different SiC contents.

**Figure 10:**
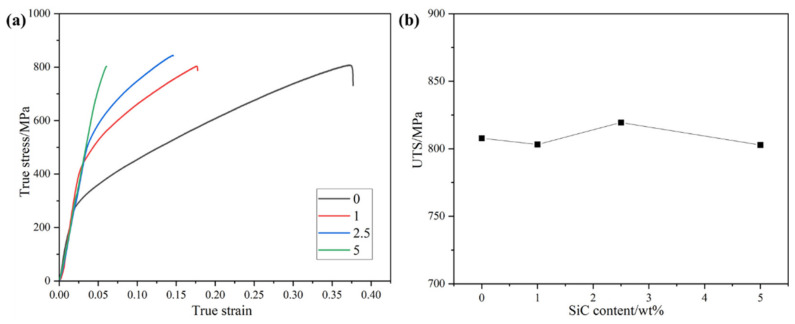
(**a**) True stress–strain curve and (**b**) strength trend graph of samples with different SiC contents.

Figure 7 shows the engineering stress–strain curves and hardness changes of the S1–S4 samples. As can be seen in Figure 7a, with the increase in SiC content, the sample elongation decreases from 46% to 6%, and the ultimate tensile strength of the samples increases from 557.9 MPa to 755.7 MPa. The hardness of the samples increases with the rise in initial SiC addition, as shown in Figure 7b, from 198.8 HV to 321.7 HV. Figure 8 shows the trend of the ultimate tensile strength and yield strength of the samples. From Figure 8a, it can be seen that with the increase in SiC content, sample strength first increases significantly, followed by a leveling-off. From Figure 8b, it can be seen that with the increase in SiC content, the yield strength of the samples increases dramatically; there is a proportional relationship between SiC content and yield strength of the samples as the latter increases from 242.5 MPa to 673.4 MPa. Figure 9 shows the elongation trend, from which it can be seen that when SiC particles are added to the samples, sample elongation decreases substantially and then tends to level off; with the continued addition of SiC, sample elongation further decreases substantially. Figure 10 shows the true stress–strain curve and strength trend graph of the samples, from which it can be seen that with the addition of SiC, the tensile strength basically remains unchanged. In conclusion, with 1% SiC addition, grain refinement occurs, and strength increases, but the strengthening effect is insufficient, with a limited improvement amplitude. At 2.5% SiC, strength is greatly improved, and plasticity/elongation does not drop as sharply as that of the 5% SiC sample. It exhibits the best comprehensive matching of strength and toughness, serving as the optimal balance point that takes hardness, strength and plasticity into account. Although the 5% SiC sample possesses the highest strength, its elongation drops to only 6%, accompanied by serious embrittlement and reduced practical application value. Consequently, the optimal comprehensive balance of strength and toughness is achieved at 2.5 wt% SiC, which can be regarded as the optimum doping content.

In polycrystalline materials, the strengthening mechanism of the material mainly originates from solid-solution strengthening, grain-boundary strengthening, dislocation strengthening and precipitation strengthening. In this study, strengthening is mainly attributed to grain-boundary strengthening, dislocation strengthening, solid-solution strengthening and dispersion strengthening. Therefore, the total strength of the sintered samples can be expressed as

σ_S1_ = σ_0_ + Δσ_gb_ + Δσ_dis_ (1)

σ_S2–4_ = σ_0_ + Δσ_gb_ + Δσ_dis_ + Δσ_D_ (2)

where σ_0_ is the intrinsic strength associated with the lattice friction strength of CoCrFeNi HEAs, and Δσ_gb_, Δσ_dis_, and Δσ_D_ denote grain-boundary reinforcement, dislocation reinforcement, and diffusion reinforcement, respectively. The lattice friction strength of HEAs is determined by their chemical composition. Under the low-energy mixing process, SiC nanoparticles exhibit no obvious chemical reaction or elemental interdiffusion with the FCC matrix of CoCrFeNi HEA. Accordingly, the intrinsic lattice friction strength of the matrix in the composite can be approximated to that of the monolithic CoCrFeNi HEA. By contrast, under the high-energy mixing process, partial decomposition and interfacial reaction of SiC occur, and small amounts of C and Si slightly dissolve into the FCC matrix, causing a minor deviation in the matrix composition. Nevertheless, the main FCC lattice structure remains unchanged, and the overall elemental composition still maintains a near-equiatomic ratio, resulting in only a slight variation in lattice friction strength without order-of-magnitude difference. Consequently, the difference in lattice friction strength between the two processes is negligible. The lattice friction strength of CoCrFeNi HEAs can be taken, which is known to be about 123 MPa from the literature [38].

The contribution of grain-boundary strengthening Δσ_gb_ can be estimated using the Hall–Petch equation given below [39].

σ_gb_ = σ_0_ + kd^−1/2^ (3)

Δσ_gb_ = σ_gb_ − σ_0_ = kd^−1/2^ (4)

where k is the strengthening factor and d is the grain size. The value of k here is 226 MPa μm^1/2^ according to Liu et al. [40]. The grain size of the samples is shown in Table 1. Substituting the individual values into the formula yields an estimate of the grain-boundary strengthening contribution shown in Table 1.

Dislocation strengthening can be calculated using the Bailey–Hirsch [41] formula given below.

(5) ∆σdis=MαGbρ12

where M is the Taylor factor (3.09). For FCC metals, the value of α is 0.2, and G is the shear modulus of the FCC phase (81 GPa) [42]. b is the Burgers vector, which is approximately 0.254 nm. ρ is the dislocation density, which can be roughly estimated from the X-ray diffraction results.

(6) $$\rho =\frac{2\sqrt{3}\varepsilon }{Db}$$

where ε is the micro-lattice strain, D is the grain size, and b is the Burgers vector. The calculated values of dislocation strengthening estimates are shown in Table 1.

According to the various strengthening contributions, the yield strength contributions of the S1–S4 samples are shown in Table 2. It can be seen that the experimental values of the yield strengths without added SiC are similar to the theoretical values, and theoretically, it is reasonable that the yield strengths obtained from the experiments are slightly lower than the theoretical values due to the presence of microstructural defects. The remaining strengthening values of the S2–S4 samples with added SiC particles can be attributed to the dispersion strengthening effect, which can be seen to increase gradually with the increase in SiC content.

**Figure 11:**
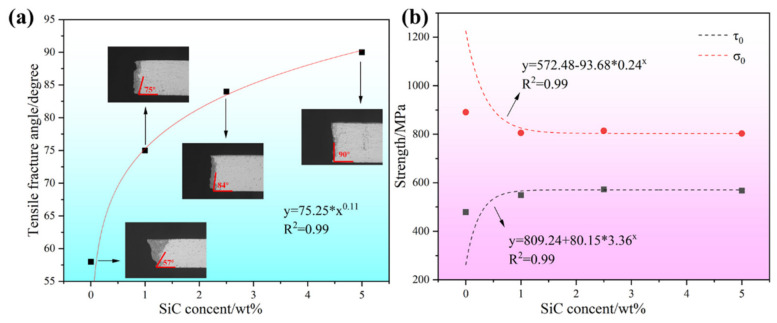
(**a**) Tensile fracture angle difference diagram and (**b**) σ_0_ and τ_0_ function diagram.

The difference in tensile fracture angle is shown in Figure 11a. With the increase in SiC content, the fracture angle gradually increases from 57° fracture with the addition of 0% SiC to 90° with the addition of 5% SiC. The change in tensile fracture angle with SiC content clearly reflects the ductile-to-brittle transition in CoCrFeNi composites. For the unreinforced alloy, the fracture angle is approximately 57°, which is close to the plane of maximum shear stress under uniaxial tension, indicating a typical ductile shear fracture mode dominated by plastic deformation. As SiC content increases to 1–2.5 wt%, the fracture angle rises to 75–84°, showing a transition from ductile to brittle behavior. The addition of SiC particles refines grains and introduces second phases, which enhance strength but restrict plastic deformation, leading to an increased tendency toward normal stress-driven fracture. At 5 wt% SiC, the fracture angle reaches 90°, perpendicular to the loading axis. This indicates a fully brittle fracture mode, where plastic deformation is severely limited, and crack propagation occurs along the plane of maximum normal stress, without significant necking. The strong correlation between fracture angle and SiC content (R^2^ = 0.99) confirms that the introduction of SiC gradually changes the dominant fracture mechanism from shear-controlled ductile fracture to normal stress-controlled brittle fracture. In the related reports on Cu-Ag alloys and Cu-Zn alloys [43,44], macroscopic samples also failed due to shear fracture at an angle of >45°, which is the same as that exhibited by the present samples.

Zhang and Eckert [45] proposed the elliptic criterion to analyze the fracture behavior of bulk metallic glasses (BMGs) by introducing parameters related to this material to explain the deformation and damage behavior of different underfills [46], expressed as follows:

(7) $${\left(\frac{\sigma }{\sigma_{0}}\right)}^{2}+{\left(\frac{\tau }{\tau_{0}}\right)}^{2}=1$$

where σ and τ are the normal and shear stresses applied on the same plane, σ_0_ and τ_0_ are the critical normal and shear fracture strengths, respectively, and α = σ_0_/τ_0_ is the fracture mode factor. When the tensile stress τ_T_ is applied to the sample, the normal stress σ and shear stress τ on any shear surface can be expressed as:

(8) $$\sigma =\sigma_{F}\;\sin^{2}\theta_{T}$$

(9) $$\tau =\sigma_{F}\;\sin\theta_{T}\cdot \cos\theta_{T}$$

Combining the above equations, the fracture mode factor α can be expressed as a function of the tensile fracture angle θ_T_, and σ_0_ and τ_0_ can be expressed as a function of the tensile fracture strength σ_F_ and α:

(10) $$\alpha =\sqrt{\frac{1}{2}}(1-\cot^{2}\theta_{T})$$

(11) $$\tau_{0}=\frac{\sigma_{F}}{2\sqrt{1-\alpha^{2}}}$$

(12) $$\tau_{0}=\frac{\sigma_{F}}{2\alpha \sqrt{1-\alpha^{2}}}$$

Without considering the effect of necking behavior, the tensile shear fracture angle is mainly determined by the parameter α and is continuously expanded from 45° to 90°, which is calculated by Equations (10)–(12) to produce the σ_0_ and τ_0_ function plots shown in Figure 11b.

Reinforcement of HEAs by addition of second-phase particles mainly relies on grain-boundary precipitation strengthening, dislocation strengthening, and dispersion strengthening. With the increase in SiC content, the alloy defects gradually increase, the critical shear stress (τ_0_) gradually increases, and the critical normal stress (σ_0_) decreases with the appearance of defects. Thus, by affecting critical shear stress (σ_0_) and normal fracture stress (τ_0_), the refinement structure and induced microdefects affect the shear fracture. In this case, an increase in α= σ_0_/τ_0_ implies that the sample will fail in a shear mode with an increased shear angle, as studied in some studies, such as in [47].

**Figure 12:**
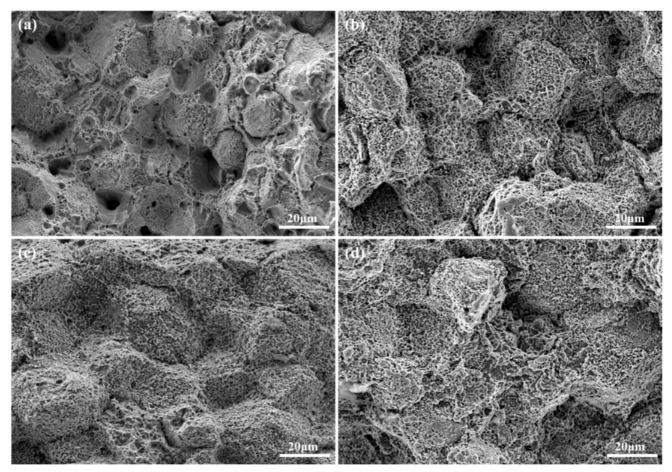
Fracture diagram of samples with different SiC contents: (**a**) 0%SiC. (**b**) 1%SiC. (**c**) 2.5%SiC. (**d**) 5%SiC.

The microscopic fracture morphology of samples S1–S4 is shown in Figure 12. When the SiC addition is 0, the fracture morphology of the samples is mainly composed of dense tough nests; with the increase in SiC content, there are plastic deformation and microporous agglomeration fracture modes in the fracture morphology, and the ductile tough nests are usually nucleated in the vicinity of the second-phase precipitates, which suggests that the precipitates act as effective barriers to the movement of dislocations and result in the stacking of dislocations at the interface between the collectives and the precipitates.

## 4. Conclusions

In this study, SiC-reinforced CoCrFeNi high-entropy alloy (HEA) composites were successfully fabricated by powder metallurgy, and the effects of SiC content on microstructure evolution and mechanical properties were systematically investigated. The obtained results largely confirmed our initial hypotheses regarding the strengthening and toughening mechanisms, while revealing some deviations that warrant discussion. The key conclusions are as follows:

(1) The unreinforced CoCrFeNi matrix exhibits a single-phase FCC structure, as expected. With the addition of SiC, a dual-phase FCC + Cr_7_C_3_ microstructure forms, driven by the decomposition of SiC and the reaction of released carbon with Cr. Contrary to our initial expectation that Cr_7_C_3_ would precipitate uniformly at the grain boundaries, increasing SiC content leads to a transition from discrete grain-boundary carbides to parallel line-like precipitates, accompanied by blurred grain boundaries. This deviation indicates that higher SiC additions induce more severe interfacial reactions and microstructural heterogeneity.
(2) Compared with the unreinforced CoCrFeNi matrix (yield strength: 242.5 MPa; ductility: 46%), the composite with 5 wt% SiC exhibits a significantly improved yield strength (673.4 MPa) and hardness (321.7 HV), albeit at the expense of ductility. This strength enhancement outperforms many other particulate-reinforced CoCrFeNi composites, such as those reinforced with Al_2_O_3_ or TiC, which typically show lower strengthening efficiency at similar reinforcement levels. The balanced combination of high strength and moderate ductility achieved in this study positions the SiC-reinforced composites as a promising alternative to conventional reinforced HEAs.
(3) For the unreinforced CoCrFeNi matrix, grain-boundary strengthening and dislocation strengthening dominate the yield strength, as expected. With SiC addition, dispersion strengthening from both unreacted SiC particles and precipitated Cr_7_C_3_ carbides becomes a primary strengthening mechanism, and its contribution increases proportionally with SiC content, as hypothesized. Additionally, SiC-induced grain refinement further enhances yield strength via the Hall–Petch effect, confirming our initial design rationale for microstructural strengthening.
(4) As predicted, the introduction of SiC induces various defects (e.g., precipitates, grain-boundary segregation, and lattice strain) that modify the critical shear stress (τ_0_) and normal fracture stress (σ_0_). These changes lead to a clear transition in fracture mode from ductile shear fracture (57° fracture angle) in the unreinforced alloy to brittle normal fracture (90° fracture angle) at 5 wt% SiC, which fully aligns with our hypothesis that increased brittleness would occur with higher SiC additions. Overall, this work demonstrates that SiC is an effective reinforcement for CoCrFeNi HEAs, offering superior strengthening efficiency compared with other ceramic reinforcements. The findings not only deepen our understanding of the microstructure–property relationships in HEA composites but also provide a practical pathway for developing high-performance materials for structural and tribological applications.

## Figures and Tables

**Table 1:** Grain size, grain-boundary strengthening and dislocation strengthening.

Simple	Size (μm)	Δσ_gb_ (MPa)	Δσ_dis_ (MPa)
S1	7.1	84.8	67.1
S2	3.1	128.4	79.9
S3	2.8	135.1	154
S4	2.3	149.0	178

**Table 2:** Theoretical and experimental values of yield strength.

Simple	Experimental Value (MPa)	Theoretical Value (MPa)
S1	242.5	274.9
S2	398.1	331.3
S3	504.2	412.1
S4	673.4	450

## Data Availability

The original contributions presented in this study are included in the article. Further inquiries can be directed to the corresponding author.
